# Notch1 is required for hypoxia-induced proliferation, invasion and chemoresistance of T-cell acute lymphoblastic leukemia cells

**DOI:** 10.1186/1756-8722-6-3

**Published:** 2013-01-05

**Authors:** Jie Zou, Peng Li, Fei Lu, Na Liu, Jianjian Dai, Jingjing Ye, Xun Qu, Xiulian Sun, Daoxin Ma, Jino Park, Chunyan Ji

**Affiliations:** 1 Department of Hematology, Qilu Hospital of Shandong University, Jinan, Shandong, 107 West Wenhua Road, Jinan, Shandong, 250012, People’s Republic of China; 2 Institute of Basic Medical Sciences, Qilu Hospital of, Shandong University,, Jinan, Shandong, Pepole’s Republic of China; 3 Otolaryngology Laboratory, Qilu Hospital, of Shandong University, Jinan, Shandong, People’s Republic of China; 4 Mary Babb Randolph Cancer Center, West Virginia University School of Medicine, Morgantown, WV, USA

**Keywords:** T-cell acute lymphoblastic leukemia, Hypoxia, HIF-1α, Notch1, Proliferation, Invasion, Chemoresistance

## Abstract

**Background:**

Notch1 is a potent regulator known to play an oncogenic role in many malignancies including T-cell acute lymphoblastic leukemia (T-ALL). Tumor hypoxia and increased hypoxia-inducible factor-1α (HIF-1α) activity can act as major stimuli for tumor aggressiveness and progression. Although hypoxia-mediated activation of the Notch1 pathway plays an important role in tumor cell survival and invasiveness, the interaction between HIF-1α and Notch1 has not yet been identified in T-ALL. This study was designed to investigate whether hypoxia activates Notch1 signalling through HIF-1α stabilization and to determine the contribution of hypoxia and HIF-1α to proliferation, invasion and chemoresistance in T-ALL.

**Methods:**

T-ALL cell lines (Jurkat, Sup-T1) transfected with HIF-1α or Notch1 small interference RNA (siRNA) were incubated in normoxic or hypoxic conditions. Their potential for proliferation and invasion was measured by WST-8 and transwell assays. Flow cytometry was used to detect apoptosis and assess cell cycle regulation. Expression and regulation of components of the HIF-1α and Notch1 pathways and of genes related to proliferation, invasion and apoptosis were assessed by quantitative real-time PCR or Western blot.

**Results:**

Hypoxia potentiated Notch1 signalling via stabilization and activation of the transcription factor HIF-1α. Hypoxia/HIF-1α-activated Notch1 signalling altered expression of cell cycle regulatory proteins and accelerated cell proliferation. Hypoxia-induced Notch1 activation increased the expression of matrix metalloproteinase-2 (MMP2) and MMP9, which increased invasiveness. Of greater clinical significance, knockdown of Notch1 prevented the protective effect of hypoxia/HIF-1α against dexamethasone-induced apoptosis. This sensitization correlated with losing the effect of hypoxia/HIF-1α on Bcl-2 and Bcl-xL expression.

**Conclusions:**

Notch1 signalling is required for hypoxia/HIF-1α-induced proliferation, invasion and chemoresistance in T-ALL. Pharmacological inhibitors of HIF-1α or Notch1 signalling may be attractive interventions for T-ALL treatment.

## Background

T-cell acute lymphoblastic leukemia (T-ALL), which accounts for 25% of cases of adult ALL, is characterized by the malignant clonal expansion of immature T-cell progenitors [1]. More than 50% of cases of T-ALL involve somatic activating mutations of Notch1 [2], a potent regulator known to play an oncogenic role in many malignancies, affecting proliferation, invasion, chemoresistance, angiogenesis and cell fate determination [3-5]. In the Notch1 signalling pathway, Notch1 transmembrane receptors become activated when ligands bind to their extracellular domains. This ligand binding results in two consecutive proteolytic cleavage events that liberate the intracellular Notch1 (ICN), which enters the nucleus and interacts with the DNA-binding protein CSL (CBF1/RBP-J, Su(H), Lag-1) to regulate expression of downstream genes [6]. In murine models, constitutive activation of Notch1 signalling induced T-ALL, demonstrating the key role of Notch1 in the pathogenesis of T-ALL [7]. Interference with aberrant Notch1 signalling in the context of T-ALL has the potential to inhibit proliferation and induce apoptosis, suggesting that Notch1 may be a pivotal oncogene [8,9].

In bone marrow, hematopoiesis occurs under relatively hypoxic conditions [10]. Hypoxia induces cells to undergo a variety of biological responses, including up-regulation of a series of physiologically important genes such as erythropoietin, glucose transporter type 1 and vascular endothelial growth factor (VEGF) [11,12]. Activation of these hypoxia-induced genes enables cells to respond to oxygen deprivation by modifying cell growth, metabolism, erythropoiesis and angiogenesis. Hypoxia-inducible factor-1 (HIF-1), a heterodimeric transcription factor consisting of HIF-1α and HIF-1β subunits, plays a critical role in cellular response to hypoxic conditions. It is well established that HIF-1α is the unique, O_2_-regulated subunit that determines HIF-1 activity [13]. Tumor hypoxia and increased HIF-1α activity can act as major stimuli for tumor aggressiveness and progression [14]. Hypoxia may also play a pivotal role in chemoresistance after leukemic chemotherapy. Severely hypoxic areas of bone marrow contain leukemic stem cells (LSCs) responsible for minimal residual disease [15,16], which results in hematologic relapse and may serve as a marker for chemoresistance [17-19]. Moreover, it has been shown that Notch signalling is augmented under hypoxic conditions in human cervical, colon, ovarian and breast cancer cell lines. These reports seem to implicate activation of the hypoxia-mediated Notch pathway in tumor cell survival and invasiveness [5,20].

We down-regulated HIF-1α and Notch1 using small interference RNA (siRNA) in order to elucidate the influence of Notch1 signalling in hypoxia and also to assess the effect of hypoxia on T-ALL proliferation, invasion and chemoresistance. We provide evidence that hypoxia-induced proliferation, invasion and chemoresistance of T-ALL cells are dependent on HIF-1α-induced functional activation of Notch1 signalling.

## Methods

### Reagents

Primary antibodies for HIF-1α and Hes1 were purchased from Abcam (Cambridge, UK), and those for Notch1 ICN, Cyclin D1, cyclin-dependent kinase 2 (CDK2), p21, MMP2, MMP9, Bcl-2, Bcl-xL, cleaved caspase-3, cleaved caspase-9 and poly (ADP)-ribose polymerase (PARP) were purchased from CST (Beverly, MA). The primary antibody for β-Actin and all secondary antibodies were obtained from Zhongshan Golden Bridge Biotech (Shenzhen, China). Sources of other reagents are indicated in the text.

### Cell culture and hypoxia

Jurkat and Sup-T1 cells were cultured in RPMI 1640 medium supplemented with 10% (v/v) heat-inactivated fetal bovine serum (FBS, Gibco, Grand Island, NY), 100 U/ml penicillin and 100 mg/ml streptomycin (Invitrogen, Carlsbad, CA) in an incubator maintained at 37°C in an atmosphere containing 5% CO_2_ and air.

Hypoxic conditions were achieved by culturing cells in a sealed, anaerobic work station (Concept 400, Ruskin Technologies, Pencoed, Wales, UK), in which the hypoxic environment (2% O_2_, 93% N_2_ and 5% CO_2_), temperature (37°C), and humidity (90%) was kept constant.

### RNA interference

The following double-strand RNA oligos specific for HIF-1α (5^′^-GUUGCCACUUCCACAUAAUTT-3^′^) and Notch1 (5^′^-UACAGUACUGACCUGUCCACUCUGG-3^′^) were synthesized by Shanghai GenePharma (Shanghai, China). Commercially available siRNA to random noncoding sequences were used for control transductions (Shanghai GenePharma). To obtain HIF-1α or Notch1 knock-down cells with transient transfection, cells were transfected with siRNA duplexes at the final concentration of 100 nM using Lipofectamine 2000 reagent (Invitrogen, Carlsbad, CA).

### Hes1 promoter luciferase assay

The luciferase assay was performed with a Notch dual-luciferase assay kit (Qiagen, Valencia, USA) following the manufacturer’s instructions. Briefly, cells (3 × 10^4^ cells/well in a 96-well plate) were transfected with a RBP-Jκ responsive firefly luciferase reporter together with an expression vector of Renilla luciferase using Lipofectamine LTX and Plus™ Reagent (Invitrogen). After 12 h, cells were washed and then cultured under hypoxic conditions for 48 h. The luciferase assay was performed with the Dual Luciferase Assay by Promega using Renilla luciferase as an internal control.

### Proliferation assay

The effect of hypoxia on the viability of T-ALL cells was evaluated by 2-(2-methoxy-4-nitrophenyl)-3-(4-nitrophenyl)-5-(2,4-disulfophenyl)-2H-tetrazolium, a monosodium salt (WST-8) assay (Dojindo Molecular Technologies, Tokyo, Japan). Briefly, cells were seeded at a density of 5 × 10^3^ cells per well in 96-well microplates and placed in the hypoxic incubator for 24 h, 48 h or 72 h, 10 ul WST-8 solution was applied to each well and they were incubated for 4 h at 37°C. Absorbance was measured at 450 nm using a microplate reader (Benchmark Microplate Reader, BIO-RAD) with a reference wavelength of 655 nm.

### Cell cycle distribution analysis

For cell cycle analysis, T-ALL cells were plated on 6-well plates and cultured under hypoxic conditions for 48 h. Cells were fixed in 70% alcohol for 1 h at 4°C, resuspended with propidium iodide (PI) solution (0.04 mg/ml) containing 0.2 mg/ml RNase A (both from Sigma-Aldrich, St. Louis, USA), and incubated at room temperature in the dark for 30 min. DNA content was then analyzed using FACS Calibur (Becton Dickinson, CA, USA).

### Drug treatment under hypoxic conditions

Jurkat and Sup-T1 cells were incubated for 24 h under hypoxic conditions, with a pulse of 1 μM dexamethasone (Sigma-Aldrich, St. Louis, USA) added for 48 h under continuous hypoxic conditions. Cells were collected for the apoptosis assay and Western analysis of Bcl-2, Bcl-xL, cleaved caspase-3, cleaved caspase-9 and cleaved PARP.

### Apoptosis assay

The apoptosis assay was performed using an Annexin V/PI Apoptosis Detection Kit (Jingmei Biotech, Shenzhen, China). Cells were harvested, resuspended in 100 μl of binding buffer, labeled with 5 μl Annexin V-biotin followed by 10 μl PI and incubated for 15 min in the dark. Then 400 μl binding buffer was added and Annexin V-PI was measured by FACS Calibur (Becton Dickinson, CA, USA).

### RNA extraction and quantitative real-time PCR

Total RNA was prepared from cell lines using TRIzol reagent (Takara, Dalian, China) according to the manufacturer’s protocol. For reverse transcription, double-stranded cDNA was synthesized from about 1 μg of total RNA using M-MLV RTase cDNA Synthesis Kit (Takara). Quantitative real-time PCR (qRT-PCR) was performed using a LightCycler 2.0 Instrument (Roche, Penzberg, Germany) with SYBR Green PCR Master Mix (Toyobo, Osaka, Japan). Samples were run in triplicate and amplified in a 20 μl reaction according to the manufacturer’s experimental protocol. The housekeeping gene β-Actin, which has relatively constant expression in T-ALL cell lines, was used as an internal control. Primer sequences were as follows (5^′^-3^′^): Notch1 forward: GGG TCC ACC AGT TTG AAT GG; Notch1 reverse: GTT TGC TGG CTG CAG GTT CT; Hes1 forward: TGA TTT GGA TGC TCT GAA GAA AGA TA; Hes1 reverse: GCT GCA GGT TCC GGA GGT; HIF-1α forward: TTT GCT GAC ACA GAA GCA AAG A; HIF-1α reverse: TTG AGG ACT TGC GCT TTC AGG; VEGF forward: GAG CCT TGC CTT GCT GCT CTA C; VEGF reverse: CAC CAG GGT CTC GAT TGG ATG; MMP2 forward: CAG GGA ATG AAT ACT GGA TCT ACT; MMP2 reverse: GCT CCA GTT AAA GGC GGC ATC CAC; MMP9 forward: GCC TGC AAC GTG AAC ATC T; MMP9 reverse: TCA AAG ACC GAG TCC AGC TT; β-Actin forward: CGG GAC CTG ACT GAC TAC CT; β-Actin reverse: AAG CAT TTG CGG TGG A.

### Western blot

Cells were harvested in ice-cold RIPA lysis buffer in the presence of the protease inhibitor phenylmethylsulfonyl fluoride (both from Beyotime, Haimen, China) for 30 min, and lysates were cleared by centrifugation. Protein concentrations in the supernatant were quantified with the bicinchoninic acid assay protein reagent kit (Sangon, Shanghai, China) according to a standardized curve. Proteins were fractionated by sodium dodecyl sulfate–polyacrylamide gel electrophoresis and transferred onto polyvinylidene fluoride membranes. Non-specific sites were blocked with 5% nonfat milk in PBS/0.1% Triton X-100 and incubated with appropriate primary antibodies overnight at 4°C, followed by incubation with horseradish peroxidase conjugated anti-mouse or anti-rabbit IgG at room temperature for 1 h. After washing, immunoreactive bands were detected by enhanced chemiluminescence (Millipore, Billerica, MA).

### Transwell invasion assay

The invasive potential of T-ALL cells was examined using transwell inserts fitted with polycarbonate filters (5 um pore size, Costar, Cambridge, MA) coated with matrigel (BD Biosciences, Bedford, MA). Matrix solutions within transwells were polymerized at 37°C for 1 h and dried onto the transwells overnight at room temperature. Cells in FBS-free medium were seeded in the upper compartment while lower wells contained 10% FBS medium. After 48 h of hypoxic incubation, the cells in the upper chamber were removed while other cells, which had passed through the filter on the underside of the membrane, were fixed with 3.7% paraformaldehyde, stained with 0.2% crystal violet, counted and captured at 100× and 400× magnification using the camera on inverted microscope. Contents of the lower compartments were collected and migrated cells were also counted. The rate of migration was expressed as the fraction of migrated cells out of the total number of cells placed in the upper compartment.

### Statistical analysis

Experiments were performed three times and data are expressed as mean ± SD. Statistical analysis was conducted using Student’s t-test. Unless otherwise specified, *P* < 0.05 was considered significant.

## Results

### Hypoxia induced accumulation of HIF-1α in T-ALL cells

**Figure 1:**
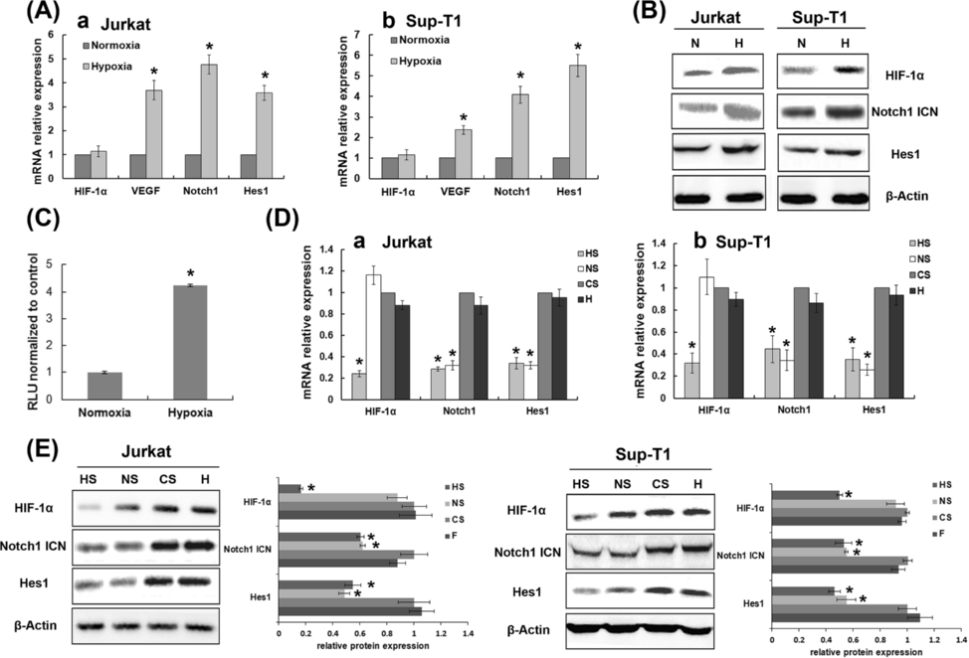
**Hypoxia potentiated Notch1 signalling via stabilization and activation of transcription factor HIF-1α.** Expression of components of the HIF-1α and Notch1 signalling pathways at the mRNA (**A**) and protein (**B**) levels in Jurkat and Sup-T1 cells cultured under hypoxic conditions for 48 h. (**A**) Results normalized to β-Actin are calibrated to a normoxic control and presented as relative mRNA expression level. *, *P* < 0.01. (**B**) N, normoxia group; H, hypoxia group. Results shown are representative of at least three independent experiments. (**C**) Notch1-luciferase-transfected Jurkat cells had significantly greater reporter gene activity when incubated in hypoxic conditions for 48 h than in normoxia. Results are calibrated to normoxic control. RLU, relative light units. *, *P* < 0.01. Components of the HIF-1α and Notch1 signalling pathways were assayed by qRT-PCR (**D**) and Western Blot (**E**) to determine the effect of knocking down HIF-1α or Notch1 expression in hypoxia. Notch1 signalling was inhibited by HIF-1α blockage, whereas HIF-1α expression was not affected by Notch1 suppression. HS, HIF-1α siRNA-transfected group; NS, Notch1 siRNA-transfected group; CS, control siRNA-transfected group. *, *P* < 0.01.

Constitutive expression of HIF-1α has been reported in many malignant human cells in vitro under normoxic conditions [21]. Normally, HIF-1α protein steadily increases in nuclei when cells are exposed to hypoxia but rapidly undergoes von Hippel–Lindau protein-mediated proteasomal degradation in the presence of oxygen [13]. Therefore, expression of HIF-1α transcription factor is regulated at the level of protein by hypoxia. We confirmed these results in T-ALL cells by showing that HIF-1α protein was constitutively expressed in the nucleus in vitro under normoxic conditions, with a further increase in HIF-1α protein but not HIF-1α mRNA under hypoxic conditions (Figure 1A-B). To test whether HIF-1α signalling was activated in hypoxia, we measured expression of the HIF-1α downstream target gene VEGF and found that expression of VEGF and HIF-1α increased concurrently (Figure 1A).

### Hypoxia and HIF-1α potentiated Notch1 signalling

To address whether hypoxia activates Notch1 in T-ALL cells, we investigated whether exogenous modulation of the Notch1 pathway in T-ALL cells was regulated differently in hypoxia than in normoxia. We found that total Notch1, activated Notch1 (Notch1 ICN) and Hes1 (a Notch1 target gene), components of the Notch1 pathway, were up-regulated in hypoxic conditions (Figure 1A-B). These results confirmed that the Notch1 pathway was more strongly activated in hypoxia than in normoxia. Analysis of Notch1 transcriptional activity using an in vitro luciferase reporter assay revealed a significant increase in Notch1 activity when T-ALL cells were exposed to hypoxic conditions (Figure 1C).

### Notch1 activation in hypoxia is HIF-1α-dependent

**Figure 2:**
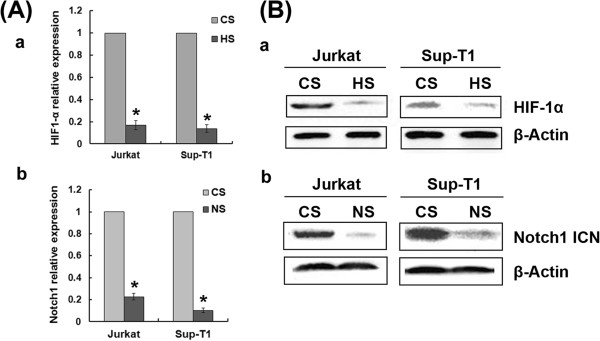
**HIF-1α and Notch1 expression are down-regulated in T-ALL cells transfected with specific siRNA.** Jurkat and Sup-T1 T-ALL cells were transfected with HIF-1α siRNA, Notch1 siRNA or control siRNA and cultured under normoxic conditions. (**A**) qRT-PCR shows mRNA levels of HIF-1α and Notch1, normalized to β-Actin and presented as relative mRNA expression level. *, *P* < 0.01. Results are an average of three independent experiments. (**B**) Western blot analysis of HIF-1α and Notch1 ICN expression in Jurkat and Sup-T1 cells transfected with HIF-1α or Notch1 siRNA. Results shown are representative of at least three independent experiments.

As demonstrated above, expression of the Notch1 target gene Hes1 was elevated under hypoxic conditions. This indicates that hypoxia enhances Notch1 activity, resulting in increased transcription of genes downstream from Notch1. To evaluate whether HIF-1α contributes to activation of hypoxia-induced Notch1 signalling, we used siRNA to silence endogenous gene expression of HIF-1α and Notch1. Under hypoxic conditions, silencing HIF-1α inhibited Notch1 signalling, as shown by decreased expression of Notch1, Notch1 ICN and Hes1, whereas silencing Notch1 did not affect HIF-1α expression (Figure 1D-E). (The effectiveness of the siRNAs designed to silence HIF-1α and Notch1 in these T-ALL cells is shown in Figure 2). These data demonstrate that hypoxia-induced Notch1 signalling activation is due to increased HIF-1α accumulation.

### Hypoxia and HIF-1α may potentially promote proliferation of T-ALL cells via activation of the Notch1 signalling pathway

**Figure 3:**
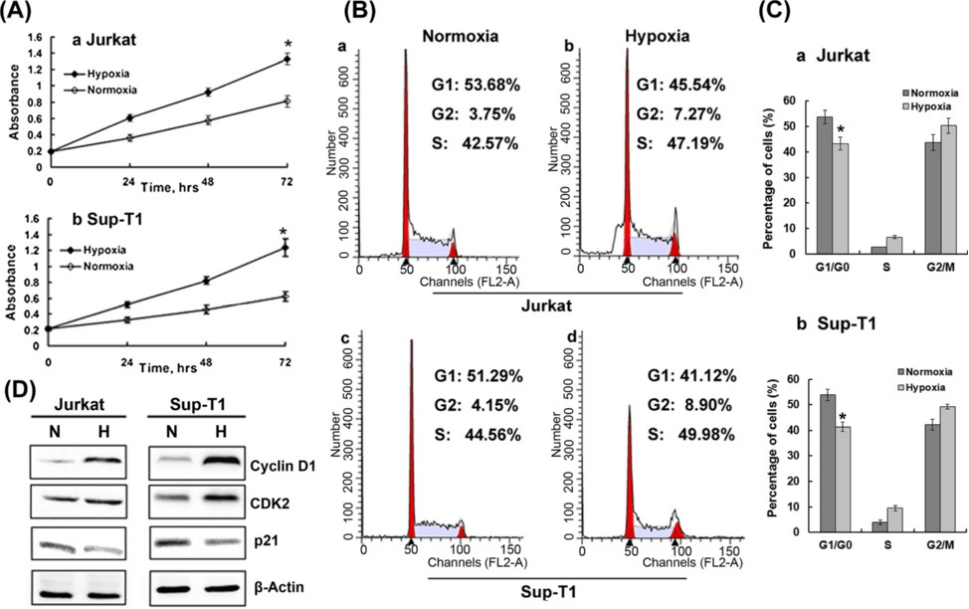
**Hypoxia promotes proliferation of T-ALL cells by reducing the G0/G1 phase of the cell cycle.** (**A**) Jurkat and Sup-T1 cells were cultured in hypoxia for up to 72 h, causing increased cell growth compared with normoxia. *, *P* < 0.01. Results are an average of three independent experiments. (**B**) After exposure to normoxia (**a**, **c**) or hypoxia (**b**, **d**) for 48 h, T-ALL cells were stained with PI and analyzed for their DNA content using FACS Calibur. The percentage of cells in G1/G0, S and G2/M in each group is shown in (**C**). *, *P* < 0.01. Data plotted are mean ± SD of three separate experiments. (**D**) Representative Western blot of cell cycle regulators Cyclin D1, CDK2 and p21 in cells cultured under hypoxic conditions for 48 h. Results shown are representative of at least three independent experiments.

**Figure 4:**
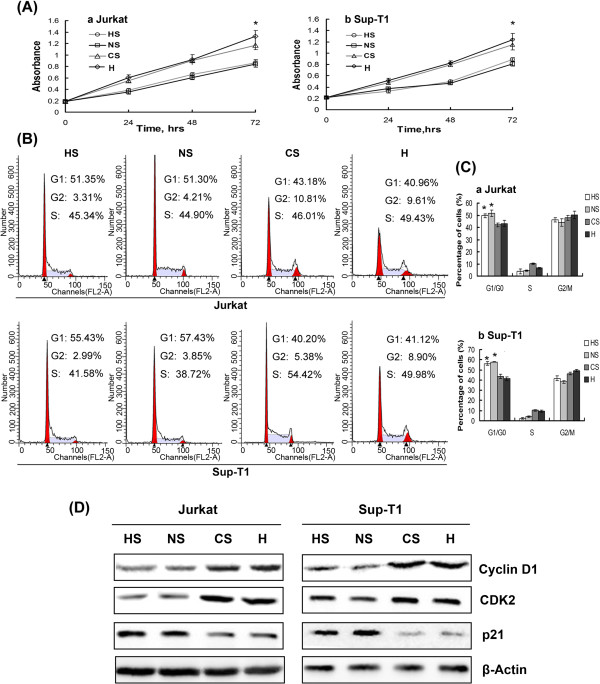
**Hypoxia/HIF-1α may potentially promote proliferation of T-ALL cells via activation of the Notch1 signalling pathway.** (**A**) Knockdown of HIF-1α or Notch1 expression significantly inhibited cell proliferation in hypoxic conditions. *, *P* < 0.01. Data plotted are mean ± SD of three separate experiments. (**B**) Similarly, cell cycle analysis showed a higher proportion of cells in the G0/G1 phase when HIF-1α or Notch1 is silenced compared with control. The percentage of cells in G1/G0, S and G2/M in each group is shown in (**C**). *, *P* < 0.01. Data plotted are mean ± SD of three separate experiments. (**D**) Representative Western blot analyses of G0/G1 phase cell cycle regulatory proteins in transfected cells cultured in hypoxia for 48 h.

To observe the effects of hypoxia and HIF-1α on growth of T-ALL cells, cell proliferation assays were performed at baseline and after 24 h, 48 h and 72 h exposure to hypoxia. As shown in Figure 3A, 2% hypoxia for 72 h led to consistently accelerated cell growth compared with normoxic conditions. We next investigated whether accelerated proliferation is mediated through regulation of the cell cycle. As shown in Figure 3B-C, 48 h exposure to hypoxia substantially reduced the number of cells in the G0/G1 phase of the cell cycle while concomitantly increasing the number of cells in the S and G2/M phases. The effect of hypoxia on cell cycle regulatory molecules operative in the G1 phase was further examined. Hypoxia reduced expression of p21 protein, the key regulator of the G1-S phase transition, while increasing protein expression of Cyclin D1 and CDK2 in T-ALL cells (Figure 3D). These results provide evidence that hypoxia promotes proliferation of T-ALL cells by reducing the number of cells in the G0/G1 phase of the cell cycle. To determine whether hypoxia-promoted cell proliferation was mediated by accumulation of HIF-1α, we knocked down HIF-1α expression under hypoxic conditions, which significantly reduced cell growth (*P* < 0.01, Figure 4A). A decreased rate of proliferation was further demonstrated by cell cycle analysis, which showed that silencing HIF-1α increased the proportion of cells in the G0/G1 phase compared with control (Figure 4B-C). When expression of G0/G1 phase cell cycle regulatory proteins was evaluated, we found that silencing HIF-1α reduced protein levels of CDK2 and Cyclin D1 while markedly elevating protein expression of p21 compared with control (Figure 4D).

To test whether hypoxia and HIF-1α promote proliferation of T-ALL cells via activation of the Notch1 signalling pathway, we silenced Notch1 signalling in T-ALL cells using Notch1 siRNA under hypoxic conditions. A viability assay showed that, after 72 h hypoxia, the number of Notch1 knockdown cells was significantly smaller than the number of control cells (*P* < 0.01, Figure 4A). This result is consistent with data from cell cycle analysis, which detected a marked G0/G1 phase arrest when Notch1 was silenced (Figure 4B-C). These experiments demonstrate that down-regulation of Notch1 expression in hypoxia reduces growth of T-ALL cells through induction of G0/G1 phase arrest. This conclusion is further supported by the protein expression pattern when Notch1 signalling is silenced under hypoxic conditions, in which CDK2 and Cyclin D1 were inhibited while p21 was increased (Figure 4D). These results indicate that increased Notch1 expression in hypoxic T-ALL cells drives cell proliferation by reducing the proportion of cells in the G0/G1 phase of the cell cycle.

### Hypoxia/HIF-1α-induced T-ALL cell invasion and MMP2/9 up-regulation require Notch1 signalling

**Figure 5:**
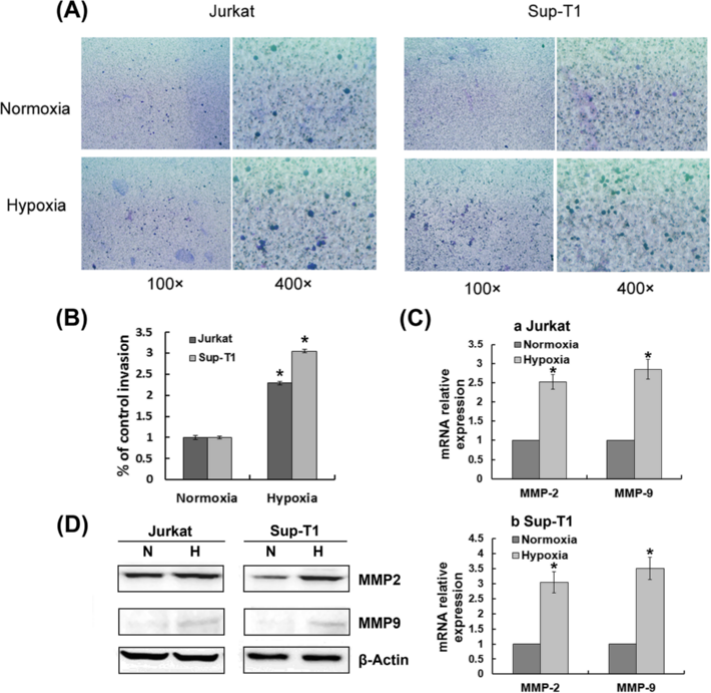
**Hypoxia promoted T-ALL invasion by transcriptionally up-regulating MMP2 and MMP9.** (**A**) 10^4^ cells were seeded onto matrigel-coated transwell inserts and allowed to migrate through the filter in normoxia or hypoxia for 48 h. Migrated cells were fixed, stained and captured at 100× and 400× magnification using the camera on an inverted microscope. (**B**) The total number of migrated cells was counted manually. Results shown are expressed as a percentage of control invasion. *, *P* < 0.01. Data plotted are mean ± SD of three separate experiments. (**C**) qRT-PCR showed that MMP2 and MMP9 were transcriptionally up-regulated in Jurkat and Sup-T1 cells kept in hypoxia for 48 h. The results normalized to β-Actin are presented as relative expression level. *, *P* < 0.01. Data plotted are mean ± SD of three separate experiments. (**D**) Western blot showing up-regulated protein levels of MMP2 and MMP9 in hypoxia. Results shown are representative of at least three independent experiments.

**Figure 6:**
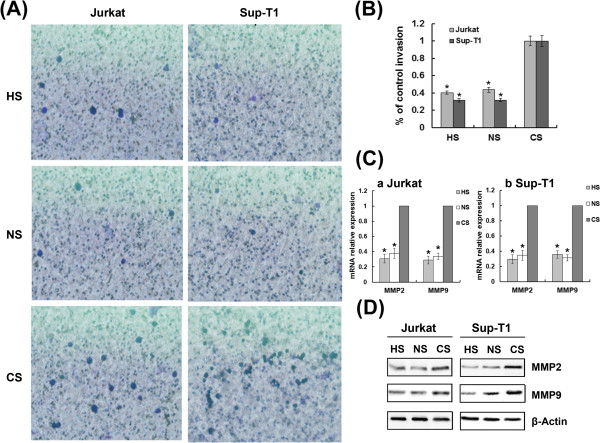
**Hypoxia/HIF-1α-induced T-ALL cell invasion and MMP2/9 up-regulation require Notch1 signalling.** (**A**) Representative 400× magnification fields in invasion assay of HIF-1α or Notch1 siRNA transfected cells exposed to hypoxia for 48 h. (**B**) The total number of migrated cells was counted manually. Results shown are expressed as a percentage of control invasion. *, *P* < 0.01. Data plotted are mean ± SD of three separate experiments. (**C**) qRT-PCR results show that inhibition of HIF-1α or Notch1 signalling attenuated the up-regulation of MMP2 and MMP9 observed during hypoxia. The results normalized to β-Actin are presented as relative expression level. *, *P* < 0.01. Data plotted are mean ± SD of three separate experiments. (**D**) Representative Western blot showing down-regulation of MMP2 and MMP9 expression by silencing HIF-1α or Notch1 in hypoxia.

To examine whether hypoxia could influence the ability of T-ALL cells to activate proinvasive protease cascades, Jurkat and Sup-T1 cells were evaluated in an invasion assay using transwell matrigel-coated chambers. We cultured both cell lines under hypoxic conditions and found that hypoxia transcriptionally up-regulated MMP2 and MMP9, and induced morphological changes typical of higher invasiveness, such as increased penetration through the matrigel-coated membrane (Figure 5). To evaluate whether HIF-1α mediates hypoxia-induced higher invasiveness, we silenced HIF-1α with specific siRNA. As illustrated in Figure 6, the increased expression of MMP2 and MMP9 induced by hypoxia was attenuated in HIF-1α knockdown cells. These results demonstrate that hypoxia/HIF-1α promotes T-ALL invasion by transcriptionally up-regulating MMP2 and MMP9.

Because hypoxia/HIF-1α promoted the activation of Notch1 and MMP2/9, and MMP2/9 genes are downstream from Notch1, we assessed whether hypoxia/HIF-1α up-regulated MMP2/9 through a Notch1-dependent mechanism. In these assays, inhibition of Notch1 signalling attenuated the up-regulation of MMP2 and MMP9 observed during hypoxia (Figure 6), and higher invasiveness was also abrogated, providing additional evidence that hypoxia/HIF-1α-induced MMP2/9 up-regulation in T-ALL cells requires Notch1 signalling.

### Notch1 signalling is required for hypoxia/HIF-1α-induced T-ALL chemoresistance

**Figure 7:**
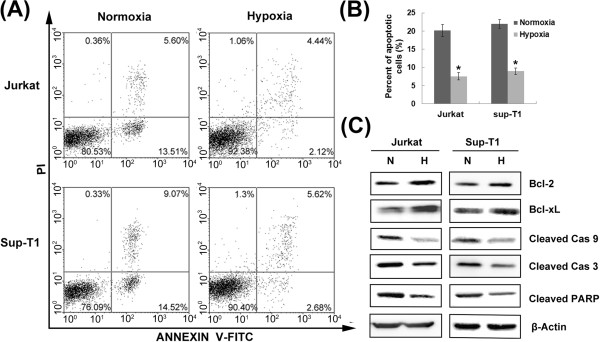
**Hypoxia resulted in significant resistance to dexamethasone in T-ALL cells.** (**A**) Jurkat and Sup-T1 cells were treated with 1 μM dexamethasone in normoxic or hypoxic conditions for 48 h and subjected to Annexin V/PI staining for flow cytometry. The percentage of cells is shown in each quadrant. (**B**) The hypoxia group had a lower percentage of apoptotic cells than the normoxia group by quantitative analysis. *, *P* < 0.01. (**C**) Antiapoptotic proteins Bcl-2, Bcl-xL and cleaved caspase 9 (Cas 9), caspase 3 (Cas 3) and PARP were estimated by Western blot. Results shown are representative of at least three independent experiments.

To clarify whether hypoxia induces chemoresistance of T-ALL cells, Jurkat and Sup-T1 cells were treated with 1 μM dexamethasone in normoxic or hypoxic conditions for 48 h and subjected to apoptosis analysis. The proportion of apoptotic cells was significantly lower in hypoxia than in normoxia (*P* < 0.01), indicating that hypoxia resulted in significant resistance to dexamethasone in T-ALL cells (Figure 7A-B).

We next assessed the effect of hypoxia on anti-apoptotic proteins, including the Bcl-2 family of anti-apoptotic proteins that plays a central role in establishing the threshold for apoptosis. As shown in Figure 7C, Western blot analysis of lysates derived from T-ALL cells subjected to dexamethasone treatment in hypoxia revealed increased expression of both Bcl-2 and Bcl-xL. Given that caspase activation has been suggested to play an important role in apoptosis, we ascertained the expression of active fragments of caspases under the same conditions. Hypoxia provoked a significant decrease in levels of the active products of caspase 3 and caspase 9, at the same time that levels of cleaved PARP, the substrate of caspase 3, dropped sharply. These results suggest that hypoxia induced chemoresistance of T-ALL cells by up-regulating Bcl-2 and Bcl-xL, which resulted in decreased dexamethasone-induced apoptosis of T-ALL cells through the intrinsic pathway.

**Figure 8:**
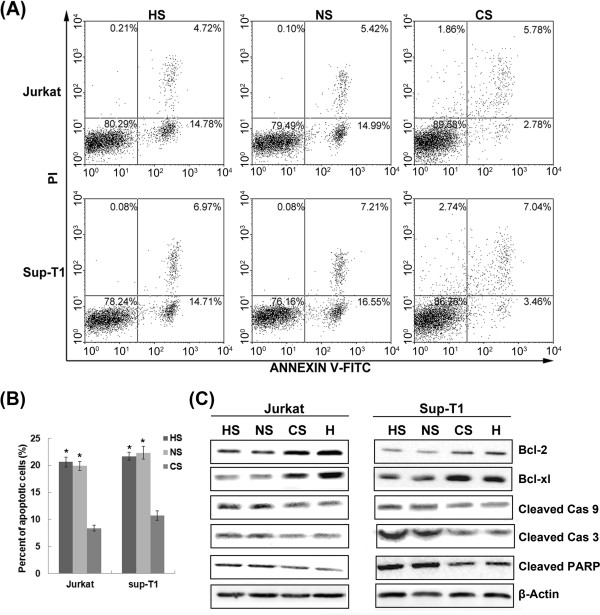
**Notch1 signalling is required for hypoxia/HIF-1α-mediated T-ALL chemoresistance.** (**A**) HIF-1α- or Notch1 siRNA-transfected cells were subjected to Annexin V/PI staining and flow cytometry after treatment with 1 μM dexamethasone under hypoxic conditions for 48 h. (**B**) Percent of total apoptotic cells by quantitative analysis. *, *P* < 0.01. Down-regulation of HIF-1α or Notch1 effectively impaired cell viability in hypoxia, with an increased proportion of apoptosis induced by dexamethasone. (**C**) Western blot analyses of apoptosis-related proteins with cell lysates under the same conditions. Results shown are representative of at least three independent experiments.

We next ascertained whether hypoxia-induced chemoresistance was mediated by HIF-1α overexpression. HIF-1α-siRNA-transfected cells showed a significantly higher rate of cell death after dexamethasone treatment than control cells (Figure 8A-B). Moreover, down-regulation of HIF-1α decreased expression of Bcl-2 and Bcl-xL and abrogated hypoxia-induced decreased caspase 3 and caspase 9 activity (Figure 8C).

It has been proposed that knockdown of Notch1 signalling with a γ-secretase inhibitor can reverse glucocorticoid resistance in T-ALL [10], implicating Notch1 activation in chemoresistance. As shown in Figure 8A-B, down-regulation of Notch1 effectively impaired cell viability in hypoxia and increased the proportion of apoptosis induced by dexamethasone. Western blot analysis of Notch1 siRNA-transfected cells showed a marked decrease in Bcl-2 and Bcl-xL at the same time that levels of activated caspase 9 and caspase 3 as well as cleaved PARP all increased, indicating activation of apoptosis (Figure 8C). These results provide evidence that Notch1 signalling is required for hypoxia/HIF-1α-mediated chemoresistance.

## Discussion

There is increasing evidence that tumor cells often reside in a low oxygen tension environment that promotes accumulation of HIF-1α and is involved in tumor progression and aggressiveness. However, the effect of hypoxia on propagation of T-ALL cells and the mechanisms by which hypoxia exerts its effects are still not clear. Notch1 signalling has been shown to play an oncogenic role in the majority of hematological malignancies including T-ALL. Further, Notch1 is related to resistance to chemotherapy, a major cause of treatment failure and poor prognosis in T-ALL. Hypoxia-induced expression of Notch receptors and ligands has been demonstrated in stem cells and malignant tumors [22,23]. In the present study, we showed that Notch1 signalling was activated by hypoxia and its transducer HIF-1α. Furthermore, we provide the first evidence that hypoxia/HIF-1α promoted the progression of T-ALL through activation of the Notch1 pathway, resulting in altered expression of downsteam genes regulating cellular proliferation, invasion and chemoresistance. HIF-1α-dependent overexpression of Notch1 in T-ALL cells is one of the major mechanisms underlying T-ALL aggressiveness and resistance to chemotherapy.

The role of hypoxia and HIF-1α in cellular proliferation has been investigated in various cell types. It has been revealed that hypoxia and HIF-1α convey stimulating or inhibiting effects on cellular proliferation and viability, depending on cell type. Studies on epithelial ovarian tumors and esophageal cancer showed that HIF-1α overexpression correlated with tumor apoptosis and patient survival [24,25]. However, in an animal model of chronic myeloid leukemia (CML), inhibition of HIF-1α impaired propagation of CML by impairing cell cycle progression and inducing apoptosis of LSCs, suggesting that HIF-1α plays a crucial role in survival maintenance of LSCs [26]. In the current study, we showed that hypoxia and HIF-1α, by reducing the proportion of cells in the G0/G1 phase of the cell cycle, promote the proliferation of T-ALL cells. Molecular investigation revealed that, under hypoxic conditions, expression of Cyclin D1 and CDK2 was increased whereas p21 expression was decreased. We also found that Notch1 ICN was augmented by hypoxia and this effect was dependent on HIF-1α accumulation, consistent with previous results [23]. Potentiation of Notch1 signalling by hypoxia was further corroborated by elevated expression of the Notch1 downstream gene Hes1 in hypoxic conditions. Inhibition of Notch1 activity using Notch1-targeted siRNA reversed hypoxia-induced changes in expression of cell cycle regulatory proteins (CDK2, CyclinD1 and p21), thus repressed the hypoxia-induced proliferation of T-ALL cells. On the basis of these results, we concluded that hypoxia potentiated Notch1 signalling in T-ALL, leading to altered expression of cell cycle regulatory proteins and increased cell proliferation.

T-ALL is characterized as leukemic cell infiltration of various organs such as lymph nodes, liver, spleen and lungs. MMPs, in particular the gelatinases MMP2 and MMP9, are an important group of zinc- and calcium-dependent proteolytic enzymes responsible for degradation of the vascular basement membrane and the extracellular matrix of lymphoid tissues [27]. A previous study suggested that hypoxia and HIF-1α overexpression contribute to tumor cell invasion and dissemination, probably through activation of MMPs [28]. Considerable evidence has accumulated that MMP9 and MMP2 play an important role in the invasiveness and propagation of several hematological malignancies [29-31]. Our studies indicate that hypoxia may be an initiating event that results in enhanced Notch1 signalling, increased expression of MMP2 and MMP9, and ultimately increased invasiveness of T-ALL. Furthermore, inhibition of Notch1 signalling abrogated hypoxia/HIF-1α-induced cell invasion, probably through down-regulation of MMP2 and MMP9. This indicates that Notch1 may serve as a critical intermediary that transforms the hypoxic response into invasion.

In addition to cellular invasion, as discussed above, chemoresistance has been recognized as another major cause of treatment failure among patients who suffer from T-ALL. Previous studies have shown that hypoxia is closely related to tumor resistance to anticancer therapy in a wide spectrum of neoplastic cells [32,33]. We hypothesized that chemoresistance to dexamethasone, an anti-leukemia drug commonly used in T-ALL treatment, was acquired by T-ALL cells in response to hypoxia. Under our hypoxic culture conditions (2% oxygen tension), dexamethasone exhibited less effectiveness. Further investigation revealed that hypoxia and HIF-1α increased expression of Bcl-2 and Bcl-xL, leading to a marked decrease in caspase activity.

Several studies have demonstrated the contribution of the Notch pathway to chemoresistance in human malignancies. Previous studies on solid tumors suggested that Notch is involved in the formation of cancer stem cells and the acquisition of the epithelial-mesenchymal transition phenotype, which are both critically associated with chemoresistance [34,35]. In myeloma and other malignant lymphoid cell lines, studies have demonstrated that Notch1 is closely related to bone marrow stroma–mediated drug resistance, and that inhibition of Notch signalling sensitizes cells to chemotherapy and prevents bone marrow-mediated chemoresistance [36,37]. Here, we showed that knockdown of Notch1 prevented the protective effect of hypoxia/HIF-1α against dexamethasone-induced apoptosis. This sensitization correlated with loss of the effect of hypoxia/HIF-1α on Bcl-2 and Bcl-xL expression.

Rohwer et al. reported that HIF-1α suppressed gastric cancer chemosensitivity via modulation of p53, indicating that hypoxia-induced chemoresistance was dependent on a functional p53 pathway [38]. Because the leukemic T-cell lines used in our study do not have functional p53, our results provide evidence that activation of the Notch1 pathway may represent an alternative mechanism for hypoxia-induced chemoresistance in mutant p53 cell lines.

HIF-1α knockdown by siRNA or antisense techniques has been shown to suppress cell growth, proliferation and migration in both normal human cells and malignant tumor cells, including umbilical vascular endothelial cells, medulloblastoma, prostate cancer and glioma [39-42]. Silencing HIF-1α also has been shown to reverse chemotherapy resistance in tumor cells [42]. Our finding, that blocking HIF-1α sensitized T-ALL cells to dexamethasone treatment, suggests that HIF-1α may be a potential target for gene therapy in T-ALL cells.

## Conclusions

The present study shows that, in T-ALL cells, proliferation, invasion and resistance to chemotherapy were stimulated when cells were exposed to hypoxic conditions, due in part to activation of Notch1 signalling. Moreover, we show that in hypoxic conditions Notch1 signalling is required to activate genes regulating cellular proliferation, invasion and chemoresistance, increasing the aggressiveness of T-ALL and its likelihood for progression. Our work is the first to characterize the interaction between Notch1 and HIF-1α in T-ALL. These results suggest that pharmacological inhibition of Notch1 or HIF-1α signalling might have potential for improving T-ALL therapy.

## Abbreviations

Cas: Caspase; CDK2: Cyclin-dependent kinase 2; CML: Chronic myeloid leukemia; CS: Control siRNA-transfected group; H: Hypoxia group; HIF: Hypoxia-inducible factor; HS: HIF-1α siRNA-transfected group; ICN: Intracelluar Notch1; LSCs: Leukemic stem cells; MMP: Matrix metalloproteinase; N: Normoxia group; NS: Notch1 siRNA-transfected group; PARP: Poly (ADP)-ribose polymerase; PI: Propidium iodide; qRT-PCR: Quantitative real-time PCR; RLU: Relative light units; siRNA: Small interference RNA; T-ALL: T-cell acute lymphoblastic leukemia; VEGF: Vascular endothelial growth factor; WST-8: 2-(2-methoxy-4-nitrophenyl)-3-(4-nitrophenyl)-5-(2,4-disulfophenyl)-2H-tetrazolium.

## Competing interests

The authors reported no potential competing interest.

## Authors’ contributions

JZ carried out the cellular biologic studies and drafted the manuscript. PL designed the primers and performed qRT-PCR. FL carried out Western blots. NL participated in the flow cytometry. JjD designed siRNAs and performed the siRNA transfection. JjY carried out the reporter gene assay. XQ provided the reagents for research. XlS provided the analysis tools. DxM participated in design and statistical analysis of the study. JP carried out the statistical analysis. CyJ conceptualized the study, participated in its design and coordination and provided vital subjects for research. All authors read and approved the final manuscript.
